# Does different surgical approaches affect tolerance to postoperative adjuvant chemotherapy in early-stage upper gastric cancer?

**DOI:** 10.3389/fsurg.2025.1647340

**Published:** 2025-12-18

**Authors:** Shengzhe Zuo, Yongkang Zhang, Xiaofeng Liao

**Affiliations:** Department of General Surgery, Xiangyang Central Hospital Affiliated to Hubei University of Arts and Science, Xiangyang, Hubei, China

**Keywords:** gastric neoplasms, proximal gastrectomy, double-tract reconstruction, total gastrectomy, Roux-en-Y reconstruction, chemotherapy tolerance

## Abstract

**Objective:**

To compare the effects of laparoscopic radical proximal gastrectomy with double-tract reconstruction (LPG-DTR) vs. laparoscopic radical total gastrectomy with Roux-en-Y reconstruction (LTG-RY) on tolerance to postoperative adjuvant chemotherapy in early-stage upper gastric cancer, providing evidence for surgical strategy selection and its impact on chemotherapy outcomes.

**Methods:**

In this retrospective cohort study, clinical data were collected from 76 patients with early-stage upper gastric cancer who underwent postoperative chemotherapy following either LPG-DTR or LTG-RY at our institution between January 2020 and January 2023. Patients were stratified into the DTR group (*n* = 35) and RY group (*n* = 41) based on surgical approach and digestive reconstruction.

**Results:**

Compared with the R-Y group, the DTR group had a longer operation time, and a smaller number of lymph node dissections (all *P* < 0.05).Chemotherapy completion rates showed no significant intergroup difference (all *P* > 0.05).The DTR group demonstrated:Lower incidence of grade ≥2 adverse events (per CTCAE v5.0 criteria), Reduced requirement for granulocyte colony-stimulating factor (G-CSF)Increased utilization of antiemetics (e.g., ondansetron, azasetron) (all *P* < 0.05).

**Conclusion:**

LPG-DTR is associated with attenuated myelosuppression and decreased incidence of specific chemotherapy-related toxicities (thrombocytopenia, hepatotoxicity, peripheral neuropathy). Preservation of partial gastric function may underlie these advantages and potentially improve quality of life during adjuvant treatment.

## 1 Introduction

In recent years, the incidence of upper gastric cancer has shown an upward trend (1). As the primary treatment modality, laparoscopic radical total gastrectomy with Roux-en-Y reconstruction (LTG-RY) has emerged as an effective surgical approach for upper gastric cancer. This technique ensures negative resection margins and maximized lymph node dissection while effectively reducing reflux symptoms. However, complications such as anemia and weight loss may occur postoperatively in some patients, directly impairing quality of life (2). Consequently, driven by clinical evidence, function-preserving demands, and guideline recommendations, proximal gastrectomy has gained acceptance as a function-preserving radical procedure for early-stage upper gastric cancer (3, 4).Double-tract reconstruction (DTR), introduced by Aikou et al. in 1988 (5), has been applied for digestive reconstruction following proximal gastrectomy, significantly reducing the incidence of postoperative reflux esophagitis. Recent studies further confirm that patients with early-stage upper gastric cancer undergoing laparoscopic radical proximal gastrectomy with double-tract reconstruction (LPG-DTR) exhibit superior nutritional status compared to those receiving LTG-RY, with no statistically significant difference in reflux symptoms (6, 7).

Even for early gastric cancer, surgical intervention alone is insufficient to ensure satisfactory survival outcomes. Postoperative adjuvant chemotherapy remains necessary to achieve radical tumor control, prolong survival, and improve quality of life. According to the Guidelines for Gastric Cancer Diagnosis and Treatment (2022) issued by China's National Health Commission (8), fluoropyrimidine-platinum doublet regimens (e.g., SOX: oxaliplatin plus S-1) are recommended for adjuvant chemotherapy in selected early-stage patients. Nevertheless, no studies have compared the impact of these two surgical approaches on chemotherapy tolerance.

Adjuvant chemotherapy typically commences at approximately 4 weeks postoperatively, when patients have recovered baseline performance status, resumed oral intake, and resolved perioperative complications. Nutritional and functional status during chemotherapy critically determines tolerance to standardized protocols. Notably, clinical trials report that <50% of patients complete planned adjuvant chemotherapy due to poor nutritional status and treatment-related complications (9, 10).

This retrospective study analyzed clinicopathological and chemotherapy data from 76 early-stage upper gastric cancer patients undergoing LPG-DTR or LTG-RY at Xiangyang Central Hospital (2020–2023). It aims to compare the effects of these procedures on chemotherapy tolerance and provide evidence for surgical strategy selection.

## 2 Materials and methods

### 2.1 Study cohort

A retrospective cohort study was conducted involving 76 patients with early-stage upper gastric cancer who underwent either LPG-DTR or LTG-RY in the Department of General Surgery between January 2020 and January 2023. The cohort comprised 60 males (78.9%) and 16 females (21.1%). Based on surgical approach, patients were stratified into:LPG-DTR group (*n* = 35), LTG-RY group (*n* = 41). The study protocol was approved by the Ethics Committee of Xiangyang Central Hospital Affiliated to Hubei University of Arts and Science, with written informed consent obtained from all participants. Baseline characteristics were comparabl between groups (all *P* > 0.05) except for N-stage classification (see Table 1).

**Table 1:** Comparison of preoperative baseline data between patients in the double tract reconstruction group and the total gastrectomy with R-Y reconstruction group.

Group	Male [(%)]	Age (*x* ± *s*)	BMI (kg/m^2^, *x* ± *s*)	Charlson rating [M (IQR)]	Tumor size (cm, *x* ± *s*)	T-stage[(%)]		N-stage [(%)]	
						T1	T2	N0	N1
DTR group	92.3	65.7 ± 7.0	23.3 ± 2.3	0.73 (1)	2.6 ± 0.8	7 (20.0)	28 (80.0)	30 (85.7)	5 (14.3)
R-Y group	85.4	64.6 ± 7.2	23.3 ± 3.3	0.66 (1)	3.1 ± 1.2	10 (24.4)	31 (75.6)	25 (61.0)	16 (39.0)
*t*/*X*^2^/*Z*	*X*^2^ = 0.729	*t* = −0.616	*t* = 0.084	*Z* = 0.344	*t* = 1.771	*X*^2^ = 0.781		*X*^2^ = 5.916	
*P*	0.393	0.540	0.949	0.731	0.081	0.377		0.015	

### 2.2 Inclusion and exclusion criteria

**Inclusion criteria**: (1) Primary gastric adenocarcinoma confirmed by preoperative gastroscopy and pathology; (2) Age 20–80 years; (3) ECOG performance status 0 or 1; (4) Tumor located in upper third of stomach; (5) Clinical stage T1-2N0-1M0(The postoperative pathological findings of signet-ring cell carcinoma or lymphovascular invasion in several patients with pT1N0 disease prompted the subsequent administration of adjuvant chemotherapy, justifying this deviation from the standard protocol); (6) Preoperative assessment for R0 resection (microscopically margin-negative); (7) Surgical approach: laparoscopic total gastrectomy with Roux-en-Y reconstruction or laparoscopic proximal gastrectomy with double-tract reconstruction, without grade 3–4 postoperative complications (Clavien-Dindo classification); (8) Postoperative SOX chemotherapy (oxaliplatin + S-1).

**Exclusion criteria**: (1) Synchronous gastric cancer lesions in distal stomach (gastric antrum); (2) Neoadjuvant chemotherapy/radiotherapy; (3) Combined resection for concomitant diseases (except cholecystectomy); (4) History of malignancy or concurrent other organ tumors; (5) Previous/current gastrointestinal resection.

### 2.3 Surgical procedures

All procedures were performed by the same surgical team. The extent of gastrectomy—either laparoscopic proximal gastrectomy (LPG) or laparoscopic total gastrectomy (LTG)—was determined based on preoperative assessment and the surgeon's intraoperative evaluation, taking into account tumor location, size, lymph node metastasis, and the adequacy of proximal and distal margins.The operation began with a systematic exploration of the liver, mesentery, abdominal wall, and pelvic cavity to identify any metastatic deposits and to assess whether the tumor had penetrated the serosa.

In the DTR group, after resection of the proximal stomach, the jejunum was divided 15 cm distal to the ligament of Treitz. The distal jejunal limb was brought up anterior to the colon and anastomosed to the esophagus in a side-to-end fashion. A side-to-side reconstruction was then created between the jejunum and the gastric remnant 15 cm distal to the esophagojejunal reconstruction. Finally, a side-to-side reconstruction was performed between the proximal jejunal stump and the jejunal limb 45 cm distal to the esophagojejunal reconstruction.

In the Roux-en-Y group, following total gastrectomy, the jejunum was divided 15 cm distal to the ligament of Treitz. The distal jejunal limb was pulled up anterior to the colon and anastomosed to the esophagus in a side-to-end manner. A side-to-side reconstruction was then established between the proximal jejunum and the jejunal limb 45 cm distal to the esophagojejunal reconstruction.

### 2.4 Chemotherapy protocol

All patients received SOX: Oxaliplatin 180 mg/d IV (2–4 h) on day 1; S-1 100 mg/d orally (40 mg AM, 60 mg PM) on days 1–14. 21-day cycles. Adjuvant chemotherapy was considered completed if a patient received 4–8 cycles postoperatively; otherwise, it was defined as incomplete.Blood tests within 5–7 days post-initiation. Safety assessments post-cycle.

### 2.5 Observation indicators

Intraoperative and Postoperative Parameters assessed included operative time, intraoperative blood loss, number of lymph nodes dissection, proximal incisal margin distance, time to first feeding, time to first defecation, and postoperative changes in hemoglobin and albumin levels. The recorded postoperative complications (within 30 days of surgery) included events such as wound infection, pulmonary infection, postoperative gastroparesis, fluid accumulation or abscess, and urinary retention. Post-chemotherapy outcomes: Chemotherapy completion rate (%), incidence of chemotherapy-related adverse events (%), and usage of supportive medications (antiemetics and G-CSF in milligrams) were recorded. During chemotherapy, adverse events were documented and graded according to the Common Terminology Criteria for Adverse Events (CTCAE) version 5.0 (CTCAE v5.0), including myelosuppression (reduction in neutrophils, platelets, hemoglobin), gastrointestinal symptoms (nausea/vomiting), peripheral neurotoxicity, hand-foot syndrome, and hepatorenal toxicity [elevated serum creatinine, alanine aminotransferase [ALT], aspartate aminotransferase [AST]]. Higher grades indicate greater severity.

### 2.6 Statistical methods

Statistical analysis was performed using SPSS 27.0 software with graphical visualization. Normally distributed continuous data are presented as mean ± standard deviation (mean ± SD) and compared using Student's *t*-test. Non-normally distributed continuous data are expressed as median with interquartile range (M[IQR]) and analyzed by Mann–Whitney *U* test. Categorical variables are described as frequencies and percentages (*n*[%]), with intergroup comparisons for non-ordinal categorical data conducted using *χ*^2^ test, Yates' corrected *χ*^2^ test, or Fisher's exact test as appropriate. Ordinal categorical data were compared using Mann–Whitney *U* test.

## 3 Results

### 3.1 Comparison of intraoperative and postoperative outcomes

Both procedures were successfully completed and achieved R0 resection in all cases. Compared to the R-Y group, the DTR group required a significantly longer operative time and yielded a fewer number of retrieved lymph nodes, with both differences being statistically significant (all *P* < 0.05). No statistically significant differences were observed in other intraoperative outcomes between the two groups (all *P* > 0.05) (see Table 2).

**Table 2:** Comparison of intraoperative and postoperative conditions between between the double-tract reconstruction group and roux-en-Y reconstruction groups.

Group	Operation time	Amount of bleeding	Proximal incisal margin	Lymph node dissection
	(min, *x* ± *s*)	[mL, M (IQR)]	[cm, M (IQR)]	(*x* ± *s*)
DTR group	319.4 ± 54.6	51.5 (5.0)	3.1 (1.0)	26.9 ± 3.9
R-Y group	271.1 ± 64.6	85.4 (15.0)	3.4 (1.8)	35.6 ± 8.6
*t*/*X*^2^/*Z*	*t* = 3.16	*Z* = −1.589	*Z* = −1.208	*t* = −5.381
*P*	0.021	0.112	0.265	<0.01

Group	Defecation time	Feeding time	Albumin decline	Hemoglobinemia
	[d, M (IQR)]	(d, *x* ± *s*)	(g/L, *x* ± *s*)	(g/L, *x* ± *s*)
DTR group	6.7 (3.0)	9.5 ± 3.7	6.4 ± 3.6	12.9 ± 4.2
R-Y group	7.6 (3.0)	9.5 ± 2.1	6.7 ± 1.9	13.4 ± 3.1
*t*/*X*^2^/*Z*	*Z* = −1.326	*t* = 0.051	*t* = 0.267	*t* = 0.158
*P*	0.185	0.959	0.790	0.875

### 3.2 Postoperative complications

No mortality occurred within 30 days postoperatively in either the DTR group or the R-Y group. There were no statistically significant differences in the incidence of early postoperative complications between the two groups (all *P* > 0.05) (see Table 3).

**Table 3:** Comparison of postoperative complications (clavien-dindo classification) between the double-tract reconstruction group and Roux-en-Y reconstruction groups.

Group	Total number of cases (%)	Wound infection	Pulmonary infection	Postoperative gastroparesis	Fluid accumulation or abscess	Urinary retention
DTR group	8 (22.9)	1	2	1	2	2
R-Y group	11 (27.8)	2	3	3	1	2
*t*/*X*^2^/*Z*	0.118					
*P*	0.731					

### 3.3 Overall chemotherapy completion

The mean time to chemotherapy initiation was (35.38 ± 1.7) days in the DTR group and (35.17 ± 1.3) days in the R-Y group. In the DTR group (*n* = 35), 30 patients (85.7%) completed the standardized chemotherapy regimen, while 34 patients (82.9%) completed it in the R-Y group (*n* = 41). Five patients (14.2%) in the DTR group discontinued standard chemotherapy or switched to monotherapy due to myelosuppression, intolerable gastrointestinal reactions, or peripheral neurotoxicity. Seven patients (17.1%) in the R-Y group failed to complete standard chemotherapy (see Table 4).

**Table 4:** Comparison of postoperative chemotherapy Status and related drugs between patients in the double tract reconstruction group and the R-Y reconstruction group.

Group	Initiation time	Rate of completion	Dosage of antiemetics	G-CSF dosage
	(*d*, *x* *±* *s*)	(*n*, %)	(mg, *x* *±* *s*)	[mg, M (IQR)]
DTR group	35.4 ± 8.8	30 (85.7)	158.4 ± 73.7	415.3 (800)
R-Y group	35.2 ± 8.1	34 (82.9)	120.6 ± 56.6	843.0 (600)
*t*/*X*^2^/*Z*	*t* = −0.102	*X*^2^ = 0.033	*t* = −2.378	*Z* = 15.01
*P*	0.919	0.856	0.02	<0.01

### 3.4 Usage of supportive medications during chemotherapy

Compared with the R-Y group, the DTR group required significantly less granulocyte colony-stimulating factor (G-CSF) (*P* < 0.05) but significantly more antiemetics (e.g., ondansetron, azasetron) (*P* < 0.05) during chemotherapy (see Table 3).

### 3.5 Incidence of adverse events during chemotherapy

Compared with the R-Y group, the DTR group exhibited significantly lower incidence rates of thrombocytopenia (*P* < 0.05), liver dysfunction (*P* < 0.05), and peripheral neuropathy (*P* < 0.05), while no statistically significant differences were observed in other adverse events between the two groups (all *P* > 0.05) (see Table 5).

**Table 5:** Comparison of the incidence of adverse events of postoperative chemotherapy between the double tract reconstruction group and the R-Y reconstruction group of patients.

Chemotherapy adverse events	CTCAE1–2		CTCAE3–4		*X* ^2^	*P*
	DTR group	R-Y group	DTR group	R-Y group		
Thrombopenia	4	16	0	0	5.028	0.039
Neutropenia	12	25	1	1	1.177	0.278
Anaemia	20	37	0	1	3.398	0.065
Abnormal liver function	8	26	0	0	6.784	0.009
Abnormal renal function	7	14	0	0	0.386	0.535
Vomiting	9	11	1	0	1.000	0.317
Peripheral neuropathy	3	14	0	0	4.297	0.038
Hand-foot syndrome	1	1	0	0	0.109	0.742

## 4 Discussion

Current research increasingly explores the clinical outcomes of radical total gastrectomy with Roux-en-Y reconstruction (LTG-RY) vs. radical proximal gastrectomy with double-tract reconstruction (LPG-DTR). A Korean randomized controlled trial (11–13) and a Chinese retrospective cohort study comparing LPG-DTR and LTG-RY in early-stage upper gastric cancer demonstrated that the DTR group exhibited less hemoglobin reduction and superior vitamin B12 absorption at 1- and 2-year postoperative intervals. The DTR group presented with earlier clinical N stages than the LTG-RY group, a finding reflective of the inherent selection bias in surgical decision-making. Specifically, radical proximal gastrectomy (facilitating double-tract reconstruction) was a viable option for earlier-stage tumors where distal stomach preservation was feasible. For more advanced tumors, however, total gastrectomy (necessitating Roux-en-Y reconstruction) was mandated to secure a sufficient distal margin and achieve oncological radicality. The DTR group required the construction of both an esophagojejunal Roux-en-Y reconstruction and a gastrojejunal reconstruction, while preserving the right gastroepiploic and right gastric vessels. This more complex procedure resulted in a longer operative time and a lower lymph node yield. Nevertheless, all patients in both groups successfully met the criteria for D2 lymphadenectomy, demonstrating that both techniques are reliable in terms of both surgical safety and oncological radicality.

Nevertheless, as adjuvant chemotherapy remains integral to comprehensive cancer management, international focus centers on whether different regimens confer long-term survival benefits. Notably, no prior studies have examined how surgical approaches affect chemotherapy completion rates and tolerance. A large-scale Korean retrospective cohort study revealed that initiating adjuvant chemotherapy within 5 weeks post-gastrectomy is critical for early gastric cancer patients; delays beyond 5 weeks correlate with significantly reduced 3- and 5-year disease-free survival (DFS) and overall survival (OS) (14). In our study, both groups initiated chemotherapy at approximately 5 weeks postoperatively, thereby eliminating timing bias in adjuvant treatment efficacy. According to our institutional data, from 2020 to 2023, a total of 251 radical resections for upper gastric cancer (including both LPG and LTG) were performed at our center. Among these, 87 patients underwent LPG with double-tract reconstruction (LPG-DTR), and 164 underwent LTG with Roux-en-Y reconstruction (LTG-RY). Postoperative complications occurred in 18 patients (20.7%) in the DTR group, including 13 cases of Grade I–II complications and 5 cases of Grade III–IV complications. In the RY group, 38 patients (23.2%) experienced postoperative complications, with 26 cases classified as Grade I–II and 12 as Grade III–IV. Statistical analysis of Grade III–IV complications yielded *χ*^2^ = 0.222, *P* = 0.638, indicating no significant difference between the two groups. Therefore, we believe our study is not subject to a selection bias where an excess of severe complications in one group led to the exclusion of significantly more patients from subsequent adjuvant therapy.

Notably, while hemoglobin and renal function changes during chemotherapy were comparable between groups, significant differences emerged in thrombocytopenia, hepatic dysfunction, and G-CSF usage. The SOX regimen (oxaliplatin + S-1) commonly induces gastrointestinal toxicities (diarrhea, nausea/vomiting, mucositis), hematological complications (neutropenia, thrombocytopenia), and neurological symptoms (acute/cumulative peripheral sensory neuropathy) (15) G-CSF—primarily used to prevent/treat chemotherapy-induced leukopenia—stimulates neutrophil/monocyte maturation and mobilizes mature cells into peripheral blood (16, 17). Prior studies confirm G-CSF safety in cervical/small-cell lung cancer (18–20), while gastric tumor tissues exhibit higher neutrophil infiltration than non-tumor tissues, correlating positively with progression and negatively with survival (21). Clinically, G-CSF predominantly manages chemotherapy-induced neutropenia.

Although neutropenia incidence showed no intergroup difference, increased G-CSF usage in the R-Y group indirectly reflects more severe neutrophil decline. Studies indicate superior vitamin B12 absorption (9) and reduced deficiency in LPG-DTR patients. Since vitamin B12 deficiency promotes ineffective megaloblast proliferation during hematopoiesis (22, 23)—affecting all blood cell lineages (24, 25) [e.g., hypersegmented neutrophils (26)]—we hypothesize that higher neutrophil counts in the DTR group may relate to postoperative vitamin B12 sufficiency and improved nutritional status, though exact mechanisms require further investigation. Consequently, LPG-DTR demonstrates attenuated hematological myelosuppression during adjuvant chemotherapy, suggesting that LTG-RY patients may require prophylactic G-CSF and thrombopoietin agents to prevent severe bone marrow toxicity.

The DTR group also exhibited lower peripheral neurotoxicity incidence. Platinum agents readily induce peripheral neuropathy (27), exacerbated by 5-fluorouracil combinations. Risk factors include pre-existing diabetes, prior neuropathy, advanced age, and vitamin B12/folate deficiency (28), typically manifesting as glove-and-stocking distribution sensory dysfunction. Given vitamin B12's critical role in hematological/metabolic/neurological systems, reduced neurotoxicity in DTR group patients may likewise stem from sustained postoperative vitamin B12 levels.

Paradoxically, despite lower adverse event rates, the DTR group required more antiemetics. Vomiting—a common SOX regimen toxicity—results from chemotherapy-induced mucosal damage triggering enterochromaffin cells to release serotonin (5-HT), which activates vagal afferent 5-HT3 receptors. Ondansetron/azasetron, selective 5-HT3 antagonists, are frontline antiemetics for chemotherapy-induced nausea/vomiting (CINV) (29) via peripheral blockade of vagal 5-HT3 receptors (primary mechanism) (30, 31). We postulate that preserved distal stomach and partial vagal innervation in DTR group patients increases 5-HT-receptor binding, intensifying emetic responses and necessitating higher antiemetic dosing. Conversely, gastrectomy in LTG-RY patients removes primary 5-HT3 receptor sites, potentially attenuating GI reactions—though mechanistic validation is needed.

Thus, although LPG-DTR reduces myelosuppression, its anatomical changes may exacerbate GI toxicity, impacting tolerance. Prophylactic antiemetics could mitigate this. Chemotherapy completion rates showed no significant difference (85.7% vs. 82.9%), indicating comparable tolerance between approaches, with LPG-DTR demonstrating lower hematological toxicity but increased emetic risk, whereas LTG-RY showed reduced GI symptoms but heightened myelosuppression.

**Study limitations**: (1) Small sample size—attributable to China's high proportion of advanced gastric cancer cases and non-adherence to standardized chemotherapy; (2) Single-center design—requiring validation through multicenter prospective trials to establish evidence-based surgical selection for early-stage upper gastric cancer.

## 5 Conclusion

This study demonstrates that proximal gastrectomy with double-tract reconstruction (LPG-DTR) exerts comparable effects on adjuvant chemotherapy tolerance as total gastrectomy with Roux-en-Y reconstruction (LTG-RY). No significant difference was observed in standardized chemotherapy completion rates between groups (85.7% vs. 82.9%, *P* = 0.735), though LPG-DTR was associated with attenuated myelosuppressive reactions during treatment. However, preservation of partial gastric function in LPG-DTR may intensify chemotherapy-induced emesis, necessitating proactive antiemetic management. Consequently, LPG-DTR represents a viable surgical alternative for early-stage upper gastric cancer, offering advantages in postoperative nutritional preservation and reduced hematological toxicity, while its propensity for gastrointestinal adverse events warrants caution. Multicenter prospective clinical studies are imperative to validate these findings and establish evidence-based surgical strategies.

## Data Availability

The raw data supporting the conclusions of this article will be made available by the authors, without undue reservation.
